# Perception, attitudes, knowledge of using complementary and alternative medicine for cancer patients among healthcare professionals: A mixed‐methods systematic review

**DOI:** 10.1002/cam4.6499

**Published:** 2023-09-07

**Authors:** Bo‐Young Youn, Jie‐Won Cha, Sungsu Cho, So‐Mi Jeong, Hyo‐Jung Kim, Seong‐Gyu Ko

**Affiliations:** ^1^ Department of Preventive Medicine, College of Korean Medicine Kyung Hee University Seoul South Korea; ^2^ Department of Applied Korean Medicine, Graduate School Kyung Hee University Seoul South Korea; ^3^ Department of Korean Medicine, Graduate School Kyung Hee University Seoul South Korea; ^4^ Department of Clinical Korean Medicine, Graduate School Kyung Hee University Seoul South Korea

**Keywords:** attitude, belief, CAM, cancer, complementary medicine, perception

## Abstract

**Background:**

With the rapid increase in the prevalence of cancer worldwide, the utilization of complementary and alternative medicine (CAM) has increased among cancer patients. This review aimed to understand the perception, attitudes, and knowledge of healthcare professionals toward using CAM for cancer patients.

**Methods:**

A mixed‐methods systematic review was undertaken in four databases. Inclusion criteria were primary studies reporting perception, attitudes, and knowledge of healthcare professionals for using CAM for cancer patients were eligible. A mixed‐methods convergent synthesis was carried out, and the findings were subjected to a GRADE‐CERQual assessment of confidence.

**Results:**

Forty‐two studies were chosen. The majority of the studies were quantitative and had less than 100 participants. Most publications were from European countries, and oncology was the highest among the specialties. The review found the following themes: feasibility of having negative adverse effects, low expectations of using CAM among HCPs, potential positive effects of using CAM, specific CAM training may be helpful, no concrete regulations to promote CAM practice, and poor physician–patient communication.

**Conclusions:**

Nurses had more positive views than other professions; oncologists were concerned regarding herb–drug interactions; integration of CAM into the healthcare system was favorable; HCPs felt the need to participate in specific CAM training; and HCPs agreed that CAM education should be provided more regularly. Future studies should explore the studies views of cancer patients and details of in‐depth evidence of CAM in oncology settings.

## 1 INTRODUCTION

Cancer rates have been remarkably increasing and are becoming more prevalent in the 21st century.^1^ According to the World Health Organization, cancer was recognized as the primary global issue for cause of death, accounting for one in six deaths in 2020, along with one‐third of cancer deaths due to smoking, high body mass index, high alcohol consumption, and lack of physical activity.^2^ In addition, the number of cancer patients is projected to continue to rise due to population aging.^3^

With the rapid growth of cancer patients worldwide, a wide array of cancer treatment modalities has been introduced: chemotherapy, hormone therapy, immunotherapy, targeted therapy, radiation therapy, and photodynamic therapy.^4^ Although the aforementioned cancer therapies have shown effectiveness in treating and curing cancer, therapy‐induced short‐ and long‐term adverse effects (AEs), have always been a significant concern for patients and physicians.^5^ Thus, a newly introduced term, integrative oncology, has been increasing recognition among healthcare professionals as patients with cancer are actively seeking and using complementary and alternative medicine (CAM) therapies as part of survivorship care.^6^ The National Center for Complementary and Integrative Health defines CAM as a group of diverse medical and healthcare systems, practices, and products that are not generally considered part of conventional medicine.^7^ Cancer patients also prefer a holistic and patient‐centered approach using evidence‐based CAM interventions for any unmet symptoms and unexpected AEs during the course of conventional care treatment.^8^

As the primary objectives of integrative oncology are to reduce the side effects of conventional treatment, to improve cancer symptoms, and to improve overall quality of life, integrative oncology has been an evolving field of comprehensive cancer care among healthcare professionals that uses various CAM modalities alongside conventional cancer treatments.^9^ It also offers adequate evidence‐based interventions with conventional oncology care, which could well address the global challenges of cancer prevention, management, and treatment.^10^

Attitudes and views of patients toward certain medicines, therapies, treatments, and interventions are vital; however, seeking physician perceptions is equally important for providing accurate benefits and risks, enhancing patient involvement in treatment decision‐making, and improving treatment plans for better outcomes of patient care.^11^ More importantly, having healthcare professionals' views, attitudes, and perspectives are highly related to shared decision‐making in cancer care as decision‐making is processed, as a collaborative effort, by physicians, patients, and caregivers to improve patient satisfaction during the course of the treatments.^12^

Despite the emerging body of literature on healthcare professionals utilizing CAM modalities for cancer patients, there has been no systematic review on this topic. Hence, the objectives of this study were to summarize the overall perception and attitudes toward using CAM therapies among healthcare professionals, with the goal of providing the necessary information for those who are willing to offer combination therapies to better care the patients, to examine knowledge toward using and suggesting CAM modalities.

The research questions of the review were to establish,

1. What are the healthcare professionals' perceptions, attitudes, and perspectives on the use of CAM for cancer patients?
2. What are the barriers to use and offer CAM modalities for cancer patients?

## 2 METHODS

A mixed‐methods synthesis methodology was carried out for this study. A mixed‐methods integrated review design generally incorporates detailed evidence of qualitative and quantitative study findings that can be synthesized into one another, facilitating the integration where both findings of studies are synthesized separately and integrated at the point of findings to address the same research purpose and questions, as per Noyes et al.^13^ This systematic review followed the Preferred Reporting Items for Systematic Reviews and Meta Analyses (PRISMA) guidelines^14^ and was registered in Open Science Framework Registries (OSF, URL: https://osf.io/dvhru).

### 2.1 Eligibility criteria

Any research articles published from inception to September 2022 that dealt with the perception, attitudes, and knowledge of healthcare professionals for using CAM for cancer patients and stated the barriers to using and offering CAM modalities for cancer patients were eligible. The scope was further specified using the population, intervention, comparison, outcomes, and study design (PICOS) format.^15^, ^16^ Only peer‐reviewed original research articles written in English were considered; articles that contained original quantitative and qualitative data were considered. Studies in the “grey literature” (opinions, Ph.D. theses, conference abstracts, editorials, etc.) were not eligible. The detailed eligibility criteria based on PICOS is outlined in Table 1.

**TABLE 1:** Eligibility criteria based on PICOS.

Components	Inclusion criteria	Exclusion criteria
Population	Healthcare professionals, including medical doctors, nurses, health educators, psychologists, social workers, physiotherapists, occupational therapists, and radiotherapists	CAM practitioners, health industry representatives, and non‐healthcare professionals
Intervention	Previously experienced with CAM modalities or considered using in oncology settings at clinics, nursing homes, hospices, and hospitals	Non‐CAM modalities (conventional therapies, modalities, and medicines) and non‐cancer patients
Comparison	Any other modalities, including non‐CAM modalities (conventional therapies, modalities, and medicines)	Not applicable
Outcomes	(1) Perceptions, attitudes, and perspectives of the healthcare professionals who have used or considered offering CAM modalities when treating cancer patients for prevention, treatment, and management of cancer (2) healthcare professionals who have mentioned the barriers to using CAM modalities for cancer patients	Not applicable
Study design	Qualitative, quantitative, and mixed‐methods studies published in English	Grey literature (opinions, Ph.D. theses, conference abstracts, editorials, etc.) and non‐systematic reviews were excluded

### 2.2 Data sources and search strategy

An initial scoping search via Google Scholar was conducted by the first author (B.Y.Y.) to identify the relevant studies. The second search was conducted by two researchers (B.Y.Y., J.W.C.) on PubMed to find the necessary keywords to further develop a suitable search strategy. The final keyword strategy was also reviewed by the oncologists. A systematic literature search was conducted in the following four databases: PubMed, EMBASE, Cochrane Library, and Google Scholar. Detailed keyword search strategies are described in Supplementary File 1.

### 2.3 Study selection, data extraction, and data analysis

A mixed‐methods synthesis methodology was carried out for this study. All eligible literature from the aforementioned databases was downloaded to EndNote 20 (Clarivate Analytics, Philadelphia, USA). The duplication process and its initial screening titles and abstracts process were done via EndNote 20, and then the remaining articles were determined for full‐text review. The full‐text screening was done in duplicate by two researchers (S.C., J.W.C.). Discrepancies were resolved through discussions with the third researcher (H.J.K.). The additional articles were found by reviewing the reference lists of the finally selected articles.

For the final included studies, the following information was extracted in duplicate via Excel 2016 (Microsoft, Redmon, USA) by two researchers (S.M.J., H.J.K.): first author name and year, country, study design, study population, number of participants, specialty, and main findings. Descriptive analyses were utilized for this review. The complete extraction was reviewed by the corresponding author (S.G.K.)

### 2.4 Data synthesis

A data‐based convergent synthesis was conducted to analyze all included studies. The synthesis first extracts the findings of the relevant sections; then, the data were grouped into the aforementioned descriptive themes in the research questions of this study. In addition, the second iteration was carried out to ensure the descriptions adequately generated the “Statements of Findings” (SoFs). With the chosen SoFs, the GRADE‐CERQual assessments were applied. The assessments were expressed as minor, moderate, or substantial with regards to the following four domains: (1) methodological limitations of the included studies; (2) relevance of the included studies to the study objectives; (3) coherence of the findings; and (4) adequacy of the data contributing to the findings. Based on an overall assessment of these four domains, confidence in the evidence for each finding was assessed as high, moderate, low, or very low. Overall, the data transformation and extraction process of the findings is presented in Supplementary File 2.

## 3 RESULTS

**FIGURE 1:**
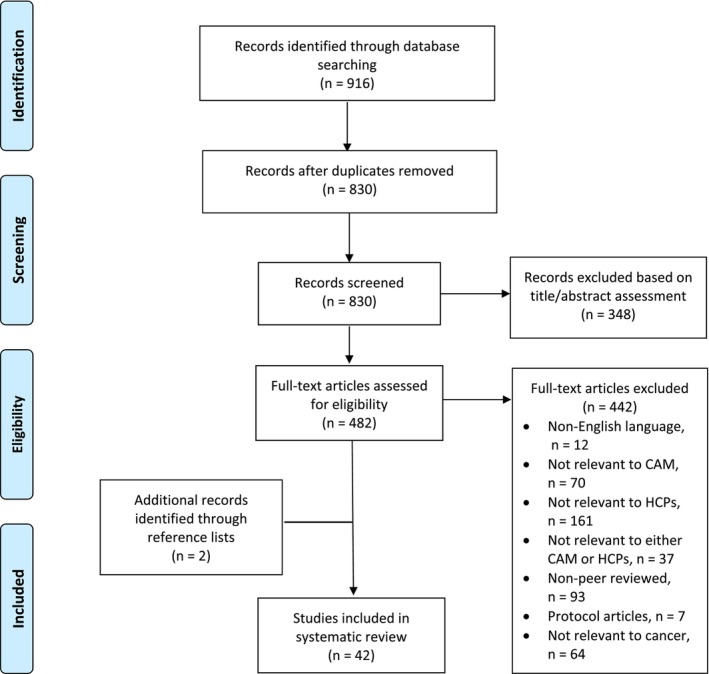
Flow diagram for the review process based on Preferred Reporting Items for Systematic Reviews and Meta‐Analyses (PRISMA).

About 916 studies were identified among the aforementioned databases, and 42 articles were eligible for this study (see Figure 1 for study selection). There were seven distinctive reasons for the articles that were excluded: non‐English language, not relevant to CAM, HCPs, and cancer, not relevant to either CAM or HCPs, protocol only, and nonpeer‐reviewed articles. Among the exclusion criteria, articles that had not relevant to HCPs were the most common.

### 3.1 Basic description of the included studies

The majority of the study design was quantitative (*n* = 34, 81%). There is a slight upward trajectory toward the number of publications, having 26 articles published between 2011 and 2022. European countries had the most publications (*n* = 16, 38%), and countries from Asia followed as the second highest (31%). Studies that recruited less than 100 participants were the most common (*n* = 18, 43%). With regards to specialties, oncology was the highest (*n* = 16, 38%), and the mixture of various specialties (*n* = 10, 26%). A detailed basic description is outlined in Table 2.

**TABLE 2:** Basic description of the included studies.

Variables	Number	Frequency
Study design		
Quantitative	34	81%
Qualitative	8	19%
Publication year		
1995–2010	16	38%
2011–2022	26	62%
Continent		
Africa	1	2%
Asia	13	31%
Europe	16	38%
Middle East	3	7%
North America	8	19%
Oceania	1	2%
Number of participants		
0–100	18	43%
101–500	15	36%
501 and over	9	21%
Specialty		
Oncology	16	38%
Pediatric oncology	3	7%
Gynecologic oncology	3	7%
Family medicine	1	2%
Palliative care	3	7%
General practice	1	2%
Mixture	11	26%
Not available	4	10%

### 3.2 Characteristics of the included studies

Of the studies, only nine articles solely had nurses as participants. With regard to the response rate, nine articles were unavailable to obtain, 16 articles mentioned less than 50%, and 17 articles indicated more than 50%. A large portion of the participants was recruited from either hospitals or medical centers (*n* = 23), and the next highest recruitment site was either medical societies or medical associations (*n* = 10). The detailed study characteristics are shown in Supplementary File 3.

### 3.3 Development of statement of findings

A detailed summary of the SoFs and its associated CERQual assessments are shown in Tables 3 and 4, respectively. Three SoFs were highlighted per research question for further review.

**TABLE 3:** Descriptive theme iteration and SoF development.

Research question	Early descriptive themes and concepts	Emergent themes	Statement of findings	Studies contributing
Q1	Safety concerns (drug–drug interaction)	Potential adverse effects using CAM	Negative adverse effects may be extremely harmful for patients	Al‐Omari 2013, Bocock 2021, Ito 2012, Klatt 2022, Lee 2008, McQuade 2012, Roth 2009, Wanchai 2015, Yang 2017, Yang 2018
	Lack of evidence			
	Harmful			
	Lack of expectations	Poor expectations	HCPs have low expectations and negative attitudes of using CAM	Alqahtani 2018, Bourgeault 1996, Christina 2019, Chang 2011, Dahlhaus 2015, Hyodo 2003, Milden 2004, Muecke 2016, Olbara 2018, Risberg 2004, Salmenpera 2003, Theodoropoulos 2005
	Lack of proven effectiveness			
	No interests			
	No exposure to CAM			
	Unnecessary			
	Delay progression	Positive attitudes and experiences	Using CAM may have positive effects	Adami 2017, Conrad 2014, Christina 2019, Crocetti 1996, Damkier 1998, McQuade 2012, Muecke 2016, Metin 2018, Kim 2016, Risberg 2004, Salmenpera 2003, Somani 2014, Wanchai 2015
	Psychological			
	Mind healing, praying			
Q2	Access to specific trainings	Access to lack of specific training, not enough opportunities for gaining experience, and training needs	Specific trainings are much needed to mention and offer CAM therapies	Al‐Omari 2013, Alqahtani 2018, Ben‐Arye 2017, King 2015, Klein 2017, Lee 2008, Lewith 2002, Milden 2004, Ott 2014, Rojas‐Cooley 2009
	Lack of opportunity			
	Lack of experienced HCPs			
	Wanting more training			
	Lack of knowledge			
	No regulations on CAM therapies	No regulation in healthcare system	There is no concrete regulation in healthcare system to use or promote CAM	Pamela 2016, Salmenpera 1998
	Not supportive			
	Expensive			
	Efficacy (shared decision making for treatment plans)	Physician–patient relationship	Poor communication by HCPs with patients (rationale/lack of explanation)	Handayani 2022, Hann 2004, Henf 2015, Roth 2009, Yang 2017, O'Beirne 2004, Grimm 2021, Rhode 2008
	Expectation			
	Lack of awareness			
	No CAM experiences			

**TABLE 4:** CERQual assessments.

Research question	Statement of findings	Studies	CerQual assessment	Explanation of assessment
Q1‐1	**Negative adverse effects may be extremely harmful for patients** A majority of HCPs were concerned of CAM being not safe for patients, especially when used with chemotherapies. HCPs have negative perspectives toward to CAM which may be harmful due to lack of scientific evidence	10 studies: Al‐Omari 2013, Bocock 2021, Ito 2012, Klatt 2022, Lee 2008, McQuade 2012, Roth 2009, Wanchai 2015, Yang 2017, Yang 2018	High confidence	Minor concerns due to adequacy, having mixed data (included patient participants)
2	**HCPs have low expectations and negative attitudes of using CAM** HCPs generally had little to no exposure of CAM so that there were no interests to consider using CAM. Due to lack of proven effectiveness and limited knowledge, HCPs are hesitant and skeptical to use and recommend CAM modalities	11 studies: Alqahtani 2018, Bourgeault 1996, Christina 2019, Chang 2011, Dahlhaus 2015, Hyodo 2003, Milden 2004, Muecke 2016, Olbara 2018, Risberg 2004, Salmenpera 2003, Theodoropoulos 2005	High confidence	Minor concerns due to adequacy, having mixed data (included patient participants)
3	**Using CAM may have positive effects** Some HCPs think that there are positive psychological effects when using CAM; in addition, CAM modalities may delay the progression and also improve overall quality of life	13 studies: Adami 2017, Conrad 2014, Christina 2019, Crocetti 1996, Damkier 1998, McQuade 2012, Muecke 2016, Metin 2018, Kim 2016, Risberg 2004, Salmenpera 2003, Somani 2014, Wanchai 2015	High confidence	Minor concerns due to adequacy, having mixed data (included patient participants)
Q2‐1	**Specific trainings are much needed to mention and offer CAM therapies** HCPs mentioned that there are no adequate training sessions to offer CAM use; however, HCPs are interested in learning more about CAM. Some HCPs believe that training regarding CAM should be mandatory in oncology. Overall, there is lack of opportunity to experience CAM	10 studies: Al‐Omari 2013, Alqahtani 2018, Ben‐Arye 2017, King 2015, Klein 2017, Lee 2008, Lewith 2002, Milden 2004, Ott 2014, Rojas‐Cooley 2009	High confidence	Minor concerns due to adequacy, lacking in‐depth data (lack of details of specific trainings)
2	**There is no concrete regulation in healthcare system to use or promote CAM** Some HCPs stated that there is no such regulations regarding CAM use. Some HCPs mentioned that there is only few regulations to cover CAM therapies whicn can get expensive for patients	2 studies: Pamela 2016, Salmenpera 1998	Low confidence	Due to low number of studies reporting on this finding
3	**Poor communication by HCPs with patients (rationale/lack of explanation)** HCPs said that lacking knowledge and awareness of CAM draw back from recommending CAM for patients. As a majority of the HCPs have never experienced CAM, HCPs cannot recommend knowing any potential positive effects	8 studies: Handayani 2022, Hann 2004, Henf 2015, Roth 2009, Yang 2017, O'Beirne 2004, Grimm 2021, Rhode 2008	Moderate confidence	Due to low number of studies reporting on this finding

### 3.4 What are the healthcare professionals' perceptions, opinions, and perspectives on the use of CAM for cancer patients?

Three SoFs were relevant to this research question: negative adverse may cause and be harmful to cancer patients (high confidence), HCPs have low expectations and negative attitudes toward using CAM (high confidence), and using CAM may have positive effects (high confidence).

#### 3.4.1 Feasibility of having negative adverse effects

Ten studies highlighted that HCPs believed that some CAM therapies pose harmful effects to patients.^17^, ^18^, ^19^, ^20^, ^21^, ^22^, ^23^, ^24^, ^25^, ^26^ Three studies mentioned that serious adverse effects could occur among patients who were undergoing chemotherapy and targeted or hormonal therapies.^24^, ^25^, ^26^ Two studies indicated that HCPs were concerned that using herbal remedies and biological supplements may cause harmful interactions combined with conventional treatments.^17^, ^21^ With regard to the physicians who also practice Kampo medicine have indicated that various adverse events were witnessed; however, the physicians were ambiguous, not knowing whether the adverse effects were truly produced due to Kampo medicine.^19^ Two studies showed that physicians, including oncologists, in China had minor concerns about having adverse effects using herbal medicines during the course of cancer treatments yet more concerned regarding Qi‐gong.^22^, ^25^

#### 3.4.2 Low expectations of using CAM among HCPs

Eleven studies generally described the high prevalence of negative perceptions of CAM that HCPs were skeptical and had no belief in CAM having institutional norms that historically have resisted CAM.^27^, ^28^, ^29^, ^30^, ^31^, ^32^, ^33^, ^34^, ^35^, ^36^, ^37^, ^38^ Two studies declared that oncologists were more negative than other nonspecialized physicians and healthcare workers.^35^, ^36^ It was notable that HCPs were more accepting of the use of CAM besides the oncology setting.^28^ One study illustrated that the essential problems are the low levels of evidence for CAM and the low levels of knowledge among HCPs toward CAM therapies.^33^ Another study pointed out that a lack of communication between HCPs and CAM practitioners may be the reason for negative attitudes toward the use of CAM.^34^

#### 3.4.3 Potential positive effects of using CAM

Thirteen studies were found to note potential positive effects when using CAM.^24^, ^29^, ^33^, ^35^, ^36^, ^39^, ^40^, ^41^, ^42^, ^43^, ^44^, ^45^ Among the chosen studies, five studies mentioned that nurses endorsed the use of CAM for positive psychological effects and also for improving quality of life.^24^, ^39^, ^42^, ^43^, ^45^ Oncologists who have positive effects regarding CAM were aligned with the previous studies that CAM therapies were likely to provide psychological and emotional support for patients.^44^ It was interesting to discover that other specialty physicians were more positive about CAM use than oncologists; moreover, young physicians were likely to have a positive view.^36^ However, oncologists have recognized that acupuncture is one of the most utilized CAM therapies.^41^ One study which surveyed Chinese physicians indicated that numerous physicians have experienced prescribing CAM, and the most famous CAM modality was Chinese herbal medicine to use for cancer patients.^22^

### 3.5 What are the barriers to use and offer CAM modalities for cancer patients?

Three SoFs were relevant to this research question: specific trainings are much needed to offer CAM therapies (high confidence), no concrete regulation in the healthcare system to use or promote CAM (low confidence), and poor communication by HCPs with patients (moderate confidence).

#### 3.5.1 Specific CAM training may be helpful

Ten studies generated insights regarding the need for specific CAM trainings for HCPs.^17^, ^21^, ^27^, ^32^, ^46^, ^47^, ^48^, ^49^, ^50^, ^51^ Despite all 10 studies generally having only a small percentage of HCPs had inadequate trainings from journals, conference presentations, seminars in medical schools, and in‐service hospital programs, a majority of HCPs expressed to participate in appropriate CAM trainings. One study mentioned that having the proper knowledge is vital for HCPs as numerous cancer patients believe that CAM is important to be part of the course of treatment so that offering proper CAM modalities for the patients may perhaps make a great difference in treatment outcomes.^27^ Among the HCPs, oncology nurses felt the strong need to have specific CAM trainings to better assist and support cancer patients, especially to improve the overall quality of life.^46^, ^50^, ^51^ On the other hand, it was surprising to find that HCPs would partake in specific CAM trainings for legal liability issues that may occur discussing and offering CAM modalities to patients.^32^

#### 3.5.2 No concrete regulations to promote CAM practice

Two studies reported that CAM practices had been sought outside of the official healthcare system.^52^, ^53^ Given the resource constraints within the healthcare system, physicians indicated a positive view of CAM integration in the current healthcare system for treating cancer patients on a broad spectrum; however, physicians also mentioned that clear scientific evidence is much needed to be integrated along with proper education for medical doctors to understand how CAM works.^52^ The other study mentioned that any changes made within the official healthcare system did not encourage patients to seek CAM to a great extent; the participants, nurses, also indicated that patients wanted to search for alternatives when concerned with vigorous examinations and overly technical treatments.^53^

#### 3.5.3 Poor physician–patient communication

Eight studies found that HCPs felt incompetent in providing adequate advice on CAM use and to initiate a conversation regarding options of CAM modalities.^23^, ^25^, ^52^, ^53^, ^54^, ^55^, ^56^, ^57^, ^58^, ^59^ All of the studies strongly suggested that knowledge on CAM should be increased by adding in‐depth information about CAM into undergraduate, graduate, and medical school curriculums, providing extra education during fellowship trainings, and increasing CAM‐focused continuing medical education. HCPs additionally stated that proper training would be valuable to provide knowledge of effectiveness and safety when using CAM for patients.

### 3.6 Quality assessment

Two researchers (J.W., M.S.J.) appraised the quality of each study independently using the Mixed‐Methods Appraisal Tool (2018 version).^60^ It has been known to be used by researchers internationally for its efficiency and reliability.^61^ Within the tool, the items 1–1 to 1–5 (for evaluation of the quantitative studies) and items 4.1 to 4.5 (for evaluation of the qualitative studies) criteria were selected for this review. Any differences were resolved by another researcher (S.G.K.) if needed (Table 5).

**TABLE 5:** Quality evaluation of included studies using the Mixed‐Methods Appraisal Tool, 2018 version.

Studies	Qualitative					Quantitative				
	1–1	1–2	1–3	1–4	1–5	4–1	4–2	4–3	4–4	4–5
Admi et al (2017)	—	—	—	—	—	Y	Y	Y	Y	Y
Al‐Omari et al (2013)	—	—	—	—	—	Y	Y	Y	Y	Y
Alqahtani et al (2018)	—	—	—	—	—	Y	Y	Y	Y	Y
Ben‐Arye (2017)	—	—	—	—	—	Y	Y	Y	N	Y
Bocock et al (2011)	—	—	—	—	—	Y	Y	Y	Y	Y
Bourgeault (1996)	Y	Y	Y	Y	Y	—	—	—	—	—
Conrad et al (2014)	—	—	—	—	—	Y	Y	Y	Y	Y
Chang et al (2011)	—	—	—	—	—	Y	Y	Y	Y	Y
Christina et al (2019)	Y	Y	Y	N	Y	—	—	—	—	—
Crocetti et al (1996)	—	—	—	—	—	Y	Y	Y	Y	Y
Dahlhaus et al (2015)	Y	Y	Y	Y	Y	—	—	—	—	—
Damkier et al (1998)	—	—	—	—	—	Y	Y	Y	Y	Y
Handayani et al (2022)	—	—	—	—	—	Y	Y	Y	Y	Y
Hann et al (2004)	—	—	—	—	—	Y	Y	Y	Y	Y
Henf et al (2015)	—	—	—	—	—	Y	C	Y	Y	Y
Hyodo et al (2003)	—	—	—	—	—	Y	Y	Y	Y	Y
Ito et al (2012)	—	—	—	—	—	Y	Y	Y	N	Y
Kim et al (2016)	—	—	—	—	—	Y	Y	Y	Y	Y
King et al (2014)	—	—	—	—	—	Y	Y	Y	Y	Y
Klatt et al (2022)	Y	Y	Y	Y	Y	—	—	—	—	—
Klein et al (2017)	—	—	—	—	—	Y	Y	Y	Y	Y
Lee et al (2008)	—	—	—	—	—	Y	Y	Y	Y	Y
Lewith et al (2002)	—	—	—	—	—	Y	Y	Y	Y	Y
McQuade et al (2012)	—	—	—	—	—	Y	C	Y	Y	Y
Milden et al (2004)	—	—	—	—	—	Y	Y	Y	Y	Y
Muecke et al (2016)	—	—	—	—	—	Y	Y	Y	Y	Y
Metin et al (2018)	—	—	—	—	—	Y	Y	Y	C	Y
O'Beirne et al (2004)	Y	Y	Y	N	Y	—	—	—	—	—
Olbara et al (2018)	—	—	—	—	—	Y	Y	Y	Y	Y
Ott et al (2015)	—	—	—	—	—	Y	Y	Y	Y	Y
Pamela et al (2016)	Y	Y	Y	Y	Y	—	—	—	—	—
Rhode et al (2008)	—	—	—	—	—	Y	Y	Y	Y	Y
Risberg et al (2004)	—	—	—	—	—	Y	C	Y	Y	Y
Rojas‐Cooley et al (2009)	—	—	—	—	—	Y	Y	Y	Y	Y
Roth et al (2009)	—	—	—	—	—	Y	Y	Y	N	Y
Salmenpera et al (1998)	—	—	—	—	—	Y	Y	Y	Y	Y
Salmenpera et al (2003)	—	—	—	—	—	Y	C	Y	Y	Y
Somani et al (2014)	—	—	—	—	—	Y	Y	Y	Y	Y
Theodoropoulos et al (2005)	—	—	—	—	—	Y	Y	Y	Y	Y
Wanchai et al (2015)	Y	Y	Y	Y	Y	—	—	—	—	—
Yang et al (2017)	—	—	—	—	—	Y	Y	Y	Y	Y
Yang et al (2018)	—	—	—	—	—	Y	C	Y	N	Y

*Note*: 1·1: is the qualitative approach appropriate to answer the research question? 1·2: are the qualitative data collection methods adequate to address the research question? 1·3: are the findings adequately derived from the data? 1·4: is the interpretation of results sufficiently substantiated by data? 1·5: is there coherence between qualitative data sources, collection, analysis, and interpretation? 4·1: is the sampling strategy relevant to address the research question? 4·2: is the sample representative of the target population? 4·3: are the measurements appropriate? 4·4: is the risk of non‐response bias low? 4·5: is the statistical analysis appropriate to answer the research question?; Y = yes. N = no. C = cannot tell.

## 4 DISCUSSION

This mixed‐methods systematic review is the first systematic review seeking perceptions and opinions of HCPs regarding CAM use for cancer patients to the best of the authors' knowledge. Due to the rapidly growing prevalence and interest in the use of CAM, it has become an increasingly important issue in the field of oncology. It is notable based on a systematic review of cancer patients using CAM (18 countries and approximately 65,000 cancer patients) that the CAM options have gained much attention in recent years and have become an important component of the course of cancer care.^62^ Additionally, according to a systematic review published in 2022, the prevalence of use of CAM was recorded to be high, indicating an increase from 24% to 71.3% over the previous 12 months.^63^ Further, it is notable that CAM is becoming more popular and accepted in Europe.^64^ These factors aligned with the basic description of the results mentioned above.

Several studies have raised concern regarding combined therapies and specifically using herbal medicines that can harm cancer patients. Clinical evidence on drug–herbal interaction may be limited in the oncology setting; however, herbal medicines have the potential as adjuvant therapy for treating chemotherapy‐induced side effects and relieving cancer symptoms.^65^ Although Qi‐gong would pose negative effectiveness, a systematic review shows that both Qi‐gong and Tai Chi are beneficial for improving quality of life.^66^ Another study also showed that Qi‐gong relieved depression and anxiety symptoms in women with breast cancer.^67^

Among the HCPs, nurses had more positive views of CAM; this factor is aligned with a few previous studies. A perspective survey in Beirut found that nurses expressed willingness to use CAM^68^; another survey of Iranian nurses implied favorable attitudes toward CAM.^69^ However, the two studies were in accordance that nurses had limited knowledge of CAM despite the positive attitudes. Observing positive views of using CAM for psychological effects, a systematic review confirmed that CAM might be effective in managing psychological symptoms, informing massage therapy has shown the most positive outcomes.^70^ Furthermore, oncologists were better known for the effectiveness of acupuncture. This result may be that acupuncture is one of the most widely used in the world^71^; two studies also indicated that acupuncture is the best known among physicians.^72^, ^73^ More importantly, acupuncture was proven to be a promising intervention after chemotherapy when applied to lung cancer patients.^74^

The negative perspectives regarding CAM could be due to having little or no specific clinical trainings. Having no trainings in therapies may believe to be unsafe or ineffective. Numerous HCPs indicated the willingness to participate in CAM trainings in order to increase knowledge of CAM, to better discuss options with patients, to learn more about evidence‐based lectures, and to consider implementing CAM into routine clinical practice for a potential synergistic effect.^75^ Conversely, HCPs are seeking to get trained for liability issues. It has known that integrating or utilizing CAM therapies may pose legal challenges as the safety of specific therapies lack evidence depending on certain conditions.^76^

Another reason for having low expectations of CAM may be HCPs having no communication with CAM practitioners, and it can be improved by including the practitioners in accountable care organizations.^77^ Oncologists, in particular, expressed negative perceptions of CAM than other specialty physicians, and this result may well associate with previous concerns about potential interactions with conventional treatment. As herbal medicines have been found to be prevalent during chemotherapy among cancer patients, the potential herb–drug interactions may be the most critical issue among oncologists and clinical pharmacologists since there is not enough evidence to decide clinical outcomes while reducing side effects.^78^ As stated by a recent publication regarding real‐world evidence of the herb–drug interactions, findings generated harmful and beneficial effects of herb‐drug interaction, yet the lack of randomized controlled trials being conducted to seek the actual interactions is currently scarce.^79^ Thus, limitations to providing strong evidence in routine clinical practice may be difficult.

HCPs had a positive view of including CAM in the healthcare system; however, there has not been much progress. Reforming healthcare delivery to include CAM for patients has been discussed for a long period of time; however, there has been a lack of appropriate evidence to inform policy makers about CAM integration so that monitoring CAM industries with more transparency should be carried out to gain more accountability.^80^ Georgia's CAM integration example has better assisted patients in ensuring the broad availability of CAM modalities, having proper regulation, financing, and coverage.^81^ A survey of health service managers further mentioned that having CAM provisions would enable patients to gain broader access to affordable CAM therapies.^82^

A majority of the HCPs have indicated that poor communication with cancer patients regarding CAM occurred due to not having enough knowledge, and HCPs further stated that education courses should be part of medical schools and continuing education programs. Based on a survey of US medical schools, it was revealed that fewer medical schools were found to include CAM courses in the curriculum and have been less enthusiastic.^83^ Further, inconsistencies in teaching and assessment in medical education have been a dilemma, advising a better curriculum design with the most up‐to‐date tools while being able to assess clinical practice in improved patient outcomes.^84^ Even though various CAM education for medical professions have been effective for improving attitudes and knowledge, evaluation for objective outcome assessments were less known.^85^

### 4.1 Limitations

This systematic review has several limitations. First, the selection was limited by including only articles in English; therefore, articles in different languages may have been missed. Some surveys were small and used nonrandom samples confined to specific groups. This limits the extent to which the findings can be generalized; a low response rate in some of the surveys increases the potential for selection bias. Lastly, preplanned convergence could not be carried out due to a low number of qualitative studies.

### 4.2 Conclusion

This review highlights the perception, attitudes, and knowledge of using CAM for cancer patients among healthcare professionals with a mixed‐method research design. There have been diverse views toward CAM. Nurses had more positive views than any other professions but did not have much knowledge; oncologists were highly concerned regarding herb–drug interactions; integration of CAM into the healthcare system seemed plausible yet lacked enough evidence; HCPs felt the strong need to participate in various CAM training to offer broader options with cancer patients; and HCPs agreed that CAM education should be provided in medical schools and continuing education programs. Clinical settings that encourage patient disclosure of CAM use may facilitate better coordination of care, reduce the risk of adverse interactions between conventional medications and CAM therapies, and lead to better patient outcomes. To resolve this discrepancy, well‐designed randomized controlled trials are needed to examine the potential benefit or adverse effect of the concomitant use of CAM. Future studies should profoundly explore the studies regarding views of cancer patients for the betterment of the cancer community.

## AUTHOR CONTRIBUTIONS

**Bo‐Young Youn:** Conceptualization (equal); methodology (equal); writing – original draft (equal); writing – review and editing (equal). **Jie‐Won Cha:** Methodology (equal); resources (equal); software (equal). **Sungsu Cho:** Formal analysis (equal); writing – review and editing (equal). **So‐Mi Jeong:** Formal analysis (equal); investigation (equal); validation (equal); writing – review and editing (equal). **Hyo‐Jung Kim:** Formal analysis (equal); writing – review and editing (equal). **Seong‐Gyu Ko:** Funding acquisition (equal); project administration (equal); supervision (equal).

## FUNDING INFORMATION

This research was supported by the National Research Foundation of Korea (NRF) grant funded by the Korean government (MIST) (no. 2020R1A5A2019413).

## CONFLICT OF INTEREST STATEMENT

The authors declare that they have no conflicts of interest.

## PROTOCOL REGISTRATION

Open Science Framework Registries (OSF, URL: https://osf.io/dvhru).

## Supporting information


Data S1

## Data Availability

Not applicable.
