# Supplementary material for: Perception, attitudes, knowledge of using complementary and alternative medicine for cancer patients among healthcare professionals: A mixed‐methods systematic review
Source: Cancer Med. 2023 Sep 7;12(18):19149–62. doi: 10.1002/cam4.6499 (PMC10557909; doi:10.1002/cam4.6499)
Supplement: Supplementary file 1 — Data S1 [file CAM4-12-19149-s001.zip › SF1_Keyword strategy.docx]

| **DB** | **Search Strategy** | |
| --- | --- | --- |
| **Pubmed** | #1 | "neoplasms"[MeSH Terms] |
|  | #2 | "cancer"[Title] OR "cancer patient*"[Title] OR "tumor*"[Title] OR "malignan*"[Title] OR "cancer care"[Title] OR "cancer treatment*"[Title] OR "oncology"[Title] |
|  | #3 | #1 OR #2 |
|  | #4 | "knowledge"[MeSH Terms] OR "perception"[MeSH Terms] OR "attitude"[MeSH Terms] |
|  | #5 | "knowledge"[Title] OR "attitude"[Title] OR "perception"[Title] OR "experience"[Title] OR "belief"[Title] OR "view"[Title] |
|  | #6 | #4 OR #5 |
|  | #7 | "medical staff*"[MeSH Terms] OR "nurses*"[MeSH Terms] OR "physicians*"[MeSH Terms] |
|  | #8 | "medical staff*"[Title] OR "nurse*"[Title] OR "physician*"[Title] OR"clinician*"[Title] OR "healthcare worker*"[Title] OR "doctor*"[Title] OR "health professional*"[Title] OR "healthcare staff*"[Title] |
|  | #9 | #7 OR #8 |
|  | #10 | "complementary therapies"[MeSH Terms] |
|  | #11 | "alternative medicine" OR "Chinese medicine"[Title] OR "complementary and alternative medicine"[Title] OR "traditional medicine"[Title] OR "Korean medicine"[Title] OR "Kampo"[TItle] OR "traditional Chinese medicine"[Title] OR "complementary medicine"[Title] |
|  | #12 | #10 OR #11 |
|  | #13 | #3 AND #6 AND #9 AND #12 |
| **Embase** | #1 | knowledge'/exp OR 'perception'/exp OR 'attitude'/exp |
|  | #2 | knowledge':ti OR 'perception':ti OR 'attitude':ti OR 'experience':ti OR 'belief':ti OR 'view':ti |
|  | #3 | #1 OR #2 |
|  | #4 | medical staff*'/exp OR 'nurse*'/exp OR 'physician*'/exp |
|  | #5 | medical staff':ti OR 'nurses':ti OR 'physicians':ti OR 'clinicians':ti OR 'healthcare workers':ti OR 'doctors':ti OR 'health professionals':ti OR 'healthcare staff':ti |
|  | #6 | #4 OR #5 |
|  | #7 | malignant neoplasm'/exp |
|  | #8 | cancer':ti OR 'cancer patient*':ti OR 'tumor*':ti OR 'malignan*':ti OR 'cancer care':ti OR 'cancer treatment*':ti OR 'oncology':ti |
|  | #9 | #7 OR #8 |
|  | #10 | alternative medicine'/exp OR 'Chinese medicine':ti OR 'complementary and alternative medicine':ti OR 'traditional medicine':ab,ti OR 'alternative medicine':ti OR 'Korean medicine':ti OR 'Kampo':ti OR 'complementary medicine':ti OR 'traditional Chinese medicine':ti |
|  | #11 | #3 AND #6 AND #9 AND #11 |
| **Cochrane** | #1 | MeSH descriptor: [Knowledge] explode all trees |
|  | #2 | MeSH descriptor: [Perception] explode all trees |
|  | #3 | MeSH descriptor: [attitude] explode all trees |
|  | #4 | #1 OR #2 OR #3 |
|  | #5 | (knowledge):ti OR (attitude*):ti OR (perception*):ti OR (experience*):ti OR (belief*):ti OR (view*):ti |
|  | #6 | #4 OR #5 |
|  | #7 | MeSH descriptor: [Medical Staff] explode all trees |
|  | #8 | MeSH descriptor: [Nurses] explode all trees |
|  | #9 | MeSH descriptor: [Physicians] explode all trees |
|  | #10 | #7 OR #8 OR #9 |
|  | #11 | ('Medical Staff*'):ti OR ('Nurse*'):ti OR ('Physician*'):ti OR (clinician*):ti OR ("healthcare worker*"):ti OR ("doctor*"):ti OR ("health professional*"):ti OR ("healthcare staff*"):ti |
|  | #12 | #10 OR #11 |
|  | #13 | MeSH descriptor: [Complementary Therapies] explode all trees |
|  | #14 | ('alternative medicine'):ti,ab,kw OR ('Chinese medicine'):ti,ab,kw OR ('complementary and alternative medicine'):ti,ab,kw OR ('traditional medicine'):ti,ab,kw OR ('Korean medicine'):ti,ab,kw OR ('complementary medicine'):ti,ab,kw OR ('traditional Chinese medicine'):ti,ab,kw OR ('Kampo'):ti,ab,kw |
|  | #15 | #13 OR #14 |
|  | #16 | #6 AND #12 AND #15 |

Google scholar

In title: mixed keywords here
